# Evaluation of sedation using pupilometry in ICUs: a pilot study

**DOI:** 10.1186/cc10936

**Published:** 2012-03-20

**Authors:** O Rouche, A Wolak-Thierry, Q Destoop, L Milloncourt, T Floch, P Raclot, J Cousson

**Affiliations:** 1 Centre Hospitalier Universitaire, Reims, France

## Introduction

The depth of hypnosis is correlated with the decrease in photomotor reflex (PMR) [1]. It would be beneficial to develop an automated, noninvasive, simple and reproducible technique allowing one to efficiently evaluate the depth of sedation in ICUs. The objective of this observational study is to evaluate the effectiveness of pupilometric video in comparison to the Bispectral index (BIS).

## Methods

Sedation level was based on the Richmond Score (RASS between -4 and -5). Exclusion criteria were neurological pathologies interfering with the PMR. Following a 320 lux flash of light, the PMR was measured by the Neurolight (IDmed). Three measurements a day were taken during 48 hours along with the collection of the BIS value (Bis Vista Anandic Medical Systems). The data collected included the variation of pupillary diameter (PD), latency time (LT) and maximal speed of pupillary constriction (V_max_). These parameters were analyzed after having classified BIS values into three groups.

## Results

A total of 186 analyses of PMR and BIS were conducted on 31 patients. The averages and standard deviations for each class of BIS were as shown in Table 1. We conducted an analysis of variance in order to compare these three groups of BIS. For the values V_max _and the PD, the ANOVA was significant. Therefore, we proceeded to compare the groups two by two using Bonferroni tests. They revealed significant difference between the BIS <40 and 40 ≤ BIS ≤ 60 group (*P *< 0.0001 for both variables) and between BIS <40 and BIS >60 (V_max _*P *< 0.0001 and PD *P *< 0.05). There was no correlation between any of the BIS groups and the LT variable.

**Table 1:** Values of V_max_, PD and TL

	BIS <40 (*n *= 68)	40 ≤ BIS ≤ 60 (*n *= 62)	BIS >60 (*n *= 37)
V_max _(mm/second)	0.98 ± 0.44	1.45 ± 0.73	1.66 ± 0.95
TL (ms)	253.8 ± 68.6	241.6 ± 41.8	240.6 ± 52.2
PD%	12.95 ± 5.58	18.3 ± 6.12	17.7 ± 6.72

## Conclusion

The V_max _and the PD seem to be relevant criteria when compared to the BIS. This noninvasive technique of monitoring the depth of sedation could be beneficial especially with patients under myorelaxant drugs. A larger study is necessary in order to confirm these results and enable one to set cut-off values for the V_max _and PD.
